# Progress in Fevers

**Published:** 1903-07-04

**Authors:** 


### PROGRESS IN FEVERS.

**Plague.**—R. Freer,[1] late surgeon to the P. and O. Steamship Company, records some reflections aroused by his observation of anti-plague methods in vogue at various ports in the Far East. The regulations of the Venice Convention, as carried out especially in Bombay, consist mainly in an examination of the crew and passengers on shore and in disinfection of the clothes and some other effects of the natives. The examination of the crews he considers thorough, as a rule, but the disinfection of clothes, etc., as he observed it, was carried out in a most incomplete and farcical manner. Methods too to prevent rats coming on board vessels were singularly incomplete and capriciously performed. If rats are prevented from getting *to* the ship in an infected part, they should be prevented from getting *from* the ship to the shore in a healthy port. Rats infest passenger ships quite as much as cargo vessels, and although it may be impossible by any practicable means to kill all the rats in a ship, the suggestion is made that much might be done to keep down the number by a vigorous system of trapping. "It should be considered as important to keep the number of rats down on board ship as to keep the brass work clean." Other suggestions made are that no native sailor should be permitted to take on board any clothes or other effects brought from his home, even if disinfected, and that a systematic plan for regulating the procedure at all ports should be adopted, instead of each port authority making its own regulations. F. Tidswell,[2] in a paper read some time ago before the British Sanitary Institute, summarised some of his conclusions gained from experience of the plague epidemic in Sydney. He formed the opinion that the disease was not spread from man to man either directly or indirectly by means of fomites. The Australian observers were convinced that rats were the source of infection for human beings, probably through the intermediation of fleas or similar vermin. Galli Valerio is quoted as asserting that fleas from rats will not bite mankind. Tidswell thinks that although they prefer their own host, they occasionally sample other hosts, and on the death of their host they seek another. There was a perfect plague of fleas at Sydney, when rats were dying in immense numbers on the wharves. R. Row[3] publishes a preliminary communication on the serum reaction of bacillus pestis in plague. A study was made of hanging drop cultures, and it was found that plague bacilli grew vigorously in normal blood serum, that the growth was scanty, and involution forms appeared in the blood serum of patients in the very early stages of the disease, and that the growth was absent and involution forms were constantly found in convalescents from plague of over six weeks. Also to a less extent this inhibitory action was observed in the serum of two patients convalescent from plague 12 and 18 months respectively. A luxuriant growth was obtained with the serum of a fatal case a few hours before death.

**Sleeping Sickness.**—Dr. Aldo Castellani,[4] a member of the commission appointed to investigate the origin of sleeping sickness in Uganda, has presented his report to the Royal Society. A summary is given of previous investigations. Cagigal and Lepierre announced the discovery of a bacillus in 1897 which, however, was not confirmed. Marchoux observed that sleeping sickness sometimes followed pneumonia and ascribes the disease to Fränkel's diplococcus, having discovered this organism in the pericardial fluid and in the nasal discharge of two cases of sleeping sickness complicated respectively by pericarditis and chronic rhinitis. The Portuguese Commission have recently constantly found a "diplo-streptococcus" present in the cerebro-spinal fluid of cases examined. This organism they found would not grow at all on gelatine and only badly on ordinary media such as agar and agar glycerine. Broden has found in the blood of all the cases examined by him a bacillus not agglutinated by the serum of the patients. Castellani does not confirm any of these discoveries, but describes a new germ found by himself post-mortem in the cerebro-spinal fluid and heart-blood of eight out of ten cases, once in the blood during life and in two cases out of three where cerebro-spinal fluid was removed by lumbar puncture. The organism is a streptococcus, varying according to its culture medium and occurring in pairs or long or short chains. It is claimed to be a distinct variety and is placed by its discoverer between streptococcus pyogenes and streptococcus lanceolatus (Fränkel's diplococcus). From the former it is differentiated by its much more vigorous growth in all media, especially agar, and its failure to coagulate milk. From Fränkel's diplococcus and from the "diplo-streptococcus" of the Portuguese it is differentiated by its vigorous growth on gelatine.

**Intermittent Fever.** — Ronald Ross[5] publishes under this heading a note explaining his method of detecting the malarial parasite in thick blood films. It consists in washing out from the film the hæmoglobin, which is opaque, and depends on the fact that even the smallest parasites adhere to the stroma of the corpuscles which contain them. The finger is pricked with a lancet-shaped needle, and a large drop of blood, about 20 c.m., spread slightly, so as to be covered by an ordinary coverslip. It is then allowed to dry, naturally, or with slight heat insufficient to fix the hæmoglobin. A watery solution of eosin is laid on with a glass rod for 15 minutes or so, according to the strength, and then washed gently off by a stream of water. The eosin solution washes out the unfixed hæmoglobin from the dried corpuscles and stains the stromata, parasites, and leucocytes. Weak methylene blue solution is then applied for a second or two and again gently washed off. The film can now be dried and mounted in Canada balsam or examined with the oil immersion. The larger pigmented parasites are visible if the hæmoglobin is simply washed out with water. The parasites can be discovered much more readily in this thicker film than in ordinary thinner films.

[1] Lancet, Jan. 24, p. 223. [2] Med. Rec., Jan. 3, p. 17. [3] Brit. Med. Jour., Dec. 20. [4] Lancet and Brit. Med. Jour., March 14. [5] Lancet, Jan. 10, p. 86.

*(To be continued.)*

